# Integrating Landscape Disturbance and Indicator Species in Conservation Studies

**DOI:** 10.1371/journal.pone.0063294

**Published:** 2013-05-01

**Authors:** Pedro Cardoso, François Rigal, Simone Fattorini, Sofia Terzopoulou, Paulo A. V. Borges

**Affiliations:** 1 Finnish Museum of Natural History, University of Helsinki, Helsinki, Finland; 2 Azorean Biodiversity Group (GBA, CITA-A) and Portuguese Platform for Enhancing Ecological Research & Sustainability (PEERS), University of the Azores, Pico da Urze, Angra do Heroísmo, Azores, Portugal; 3 Water Ecology Team, Department of Biotechnology and Biosciences, University of Milano Bicocca, Milan, Italy; 4 Department of Ecology and Taxonomy, Faculty of Biology, National and Kapodistrian University of Athens, Athens, Greece; French National Institute for Agricultural Research (INRA), France

## Abstract

Successful conservation plans are conditioned by our ability to detect anthropogenic change in space and time and various statistical analyses have been developed to handle this critical issue. The main objective of this paper is to illustrate a new approach for spatial analysis in conservation biology. Here, we propose a two-step protocol. First, we introduce a new disturbance metric which provides a continuous measure of disturbance for any focal communities on the basis of the surrounding landscape matrix. Second, we use this new gradient to estimate species and community disturbance thresholds by implementing a recently developed method called Threshold Indicator Taxa ANalysis (TITAN). TITAN detects changes in species distributions along environmental gradients using indicators species analysis and assesses synchrony among species change points as evidence for community thresholds. We demonstrate our method with soil arthropod assemblages along a disturbance gradient in Terceira Island (Azores, Portugal). We show that our new disturbance metric realistically reflects disturbance patterns, especially in buffer zones (ecotones) between land use categories. By estimating species disturbance thresholds with TITAN along the disturbance gradient in Terceira, we show that species significantly associated with low disturbance differ from those associated with high disturbance in their biogeographical origin (endemics, non-endemic natives and exotics) and taxonomy (order). Finally, we suggest that mapping the disturbance community thresholds may reveal areas of primary interest for conservation, since these may host indigenous species sensitive to high disturbance levels. This new framework may be useful when: (1) both local and regional processes are to be reflected on single disturbance measures; (2) these are better quantified in a continuous gradient; (3) mapping disturbance of large regions using fine scales is necessary; (4) indicator species for disturbance are searched for and; (5) community thresholds are useful to understand the global dynamics of habitats.

## Introduction

Habitat destruction converts previously homogeneous landscapes into a mosaic of new land use types that can be unfavorable to species persistence [1]–[3]. This mosaic usually includes areas of intensive agriculture, exotic species plantations, non-managed alien vegetation and remnant natural areas. However, border areas between different habitats create edge effects, often promoting the extinction of many disturbance sensitive species in native habitats [4]. Such transition areas are called “ecotones” and often represent diverse habitats in the ecological boundary between native vegetation and managed habitats [5]–[7]. In addition, ecotones may promote the dispersal of species through landscapes (e.g. invasive species), modify species interactions and trophic web structure and promote ecosystem change [8].

Often, only a few habitat patches and/or sites are kept relatively safe from human disturbance and it is becoming increasingly important to identify which areas still keep mostly intact communities. Detecting ecological community thresholds, i.e., points along continuous gradients where there are major changes in community composition [9]–[11], is a way to identify where and how to protect native species and their biotic interactions [12]. This is particularly important when clear anthropogenic gradients are identified, because such gradients may be explicitly modified in a conservation planning framework, for example, by selecting reserve areas where certain uses are forbidden and others encouraged. It is important to note that two different approaches can be followed when studying edge effects [13]: (1) a discrete approach where ‘edge’ is compared with ‘interior’; (2) assessing how communities change along a continuum.

A number of methods have been proposed to identify ecological thresholds [13]–[16]. Most are aggregated methods, in the sense that species-specific responses are often lost, especially for the least abundant species, which often are of special conservation concern [17]. Recently, Baker and King [18], [19] introduced a new method called Threshold Indicator Taxa ANalysis (TITAN) to identify ecological thresholds at both individual species and community level along environmental gradients. This approach revealed to be useful for different taxa and environmental gradients at different regions. However, to our knowledge, it was never tested along an anthropogenic disturbance gradient that explicitly considers a mosaic landscape configuration. In this paper, we present a new approach to identify ecological community thresholds along disturbance gradients, combining TITAN with a new disturbance metric that explicitly considers landscape configuration. To test our approach, we studied Azorean arthropods, which represent a particularly interesting model system for both theoretical and practical reasons.

Oceanic islands are home to large numbers of endemic species, either through in-situ speciation (neoendemics) or after the extinction of populations outside the islands (palaeoendemics). Such species, together with other native species (which occur anywhere else and reached the islands through their own means) create very unique communities [20]. These communities are often more prone to be disrupted by the introduction of exotic species than their continental counterparts, as they may not be able to cope with the introduction of certain, previously inexistent, ecological traits into the ecosystem [21]–[23]. Additionally, being isolated, many populations are unable to recover from past disturbance events, when rescue effects are impossible. As such, oceanic islands have been stage to most recorded extinctions worldwide, mainly driven by habitat destruction and species introductions [20], [24], [25].

The Azorean archipelago, which was mostly covered by Laurisilva forest prior to human settlement, has undergone drastic land use changes since the first inhabitants arrived almost 600 years ago [26]–[28]. Such changes are thought to have caused the extinction of numerous endemic species, particularly in the most disturbed islands, where few and minute native forest patches remain [29]. However, while some human-modified habitats, such as exotic forest plantations, may harbor some endemic and native species [26], [30], others (like intensively managed pastures) are known to be mainly dominated by exotic species [31], [32]. In particular, there is a clear dissimilarity gradient in community composition according to the disturbance level of a particular land use type [26], [33]. If and where any threshold in community composition along a disturbance gradient can be found is nevertheless still unanswered.

Arthropods are the most diverse group in the Azores as mostly elsewhere [34]. Although they provide multiple ecosystem services, they are often neglected in conservation policies and programs, even if known to be prone to the same threats as any other organisms [35]. This is caused by different reasons, the most obvious being the lack of information on which species are living (the Linnean shortfall), where they are persisting (the Wallacean shortfall), how their abundances change in space and time (the Prestonian shortfall) and how sensitive they are to ecological change (the Hutchinsonian shortfall) [35]. Because of intensive sampling during the latter decades [36], the Azorean Islands are exceptions to the rule and much is now known about their arthropod fauna, including how different species abundances change in response to anthropogenic disturbance.

As seen, the arthropods of the Azorean Islands are a particularly well suited case study to illustrate the general aims of our approach, namely, (1) to quantify a continuous disturbance gradient according to the different land uses, their perceived disturbance level and the surrounding matrix of different uses; (2) to find the species that respond to the gradient according to their abundance and spatial distribution; (3) to determine the shift point for each species responding significantly to the gradient (i.e. uneven distribution along the gradient); (4) to assign species to either negative or positive response groups (i.e. the ones whose abundances are negatively or positively correlated with disturbance); (5) to combine species information to compute community threshold(s) and; (6) to investigate whether some particular features of the selected species may be related to the direction of their response.

## Materials and Methods

### Study area

The Azorean archipelago is located in the North Atlantic, roughly between 37° to 40°N latitude and 25° to 31°W longitude. It comprises nine main islands and some small islets aligned along a roughly WNW-ESE trend. They are fully oceanic, that is, they are totally volcanic islands of recent origin (8.12–0.25 Myr for the oldest areas of the main islands). In this study we focused on Terceira Island (402 km^2^), as this island had the most information about all arthropod taxa and land use types [32], [36]. Terceira is formed by four main volcanic polygenetic complexes (Cinco Picos, Guilherme Moniz, Pico Alto and Serra de Santa Bárbara). The highest point (Serra de S. Bárbara, 1023 m) is also the most recent (0.025 Myr B.P.) of the three major island complexes. The climate is temperate oceanic, i.e. strongly influenced by the ocean and by its topography, which produces high levels of relative atmospheric humidity that can reach 95% of annual average in the native high altitude semi-tropical evergreen laurel forest, while it restricts temperature fluctuations throughout the year.

### Fieldwork

We used a dataset of arthropod species distribution and abundance in Terceira Island previously published [26]. A total of 72 sites were sampled between 2000 and 2007, between June and September, once per site: 36 in natural forests and 36 in non-native habitats, the latter including 9 in exotic forests, 11 in semi-natural pastures and 16 in intensively managed pastures. We tried to spread the sampling sites all over the island independently of the surrounding land use matrix, although intensively managed pastures tend to be located in peripheral low-altitude areas, while natural forests tend to be present only in central high-altitude areas. At each site, epigean soil fauna was captured along 150 m long transects. Thirty pitfall traps, consisting of plastic cups with a top diameter of 42 mm and 78 mm deep, were dug into the soil so that the rim of the cup was level with the surface. Half of the traps were filled with approximately 60 ml of diluted ethylene glycol (anti-freeze liquid) and the other half with the same volume of a general attractive solution (Turquin). Traps were spaced 5 m from one another, alternating Turquin and ethylene glycol traps, and were left open for two-weeks at each site [26]. All Araneae, Opiliones, Pseudoscorpiones, Diplopoda, Chilopoda and Hexapoda (excluding Collembola, Diplura, Diptera and Hymenoptera) were initially sorted into morphospecies by students using vouchered specimens under supervision of a trained taxonomist (PAVB). All unknown morphospecies were subsequently sent to several taxonomists for species identification. All species were classified as endemic (E), native non-endemic (N) or introduced/exotic (I). Endemic species refer to species found only in the Azores. Native non-endemic species arrived by long-distance dispersal to the Azores, cannot be associated with human activities (intentional or accidental human introduction) and are also known from other regions. Exotic species are those believed to have arrived in the archipelago as a consequence of human activities and often have a cosmopolitan distribution. All species were also classified as predators, herbivores, fungivores or saprophagous, according to the criteria of Moran and Southwood [37]. Fungivores are a specialized guild of beetles living in forest litter or associated with dead wood or under bark, many of the species found in Azores being endemic (e.g. Zopheridae), and although very few species were present, we kept them separate from herbivores.

### Disturbance index

We designed a new index of “landscape disturbance” (D) that reflects an anthropogenic disturbance gradient by explicitly considering landscape configuration. A land use map of 100×100 m resolution depicting the location of all land use types was built based on aerial photography [38], with native forests further delimited and confirmed by fieldwork (C. Gaspar, unpublished data). Based on previous work [26], we knew the proportion of endemic, native and exotic species typical to each land use type present in the island. With such data, it was possible to infer the disturbance level of each land use relative to an undisturbed native forest. This was used to rank the different land uses and each was given a value of “local disturbance” (*L*) as follows : Natural forests = 0, Natural(ized) vegetation or rocky outcrops = 1, Exotic forests = 2, Semi-natural pastures = 3; Intensively managed pastures = 4; Orchards/agriculture areas = 5; Urban/industrial areas = 6. Different scales for L values were tested with similar results and we chose to present and discuss the simplest case. To the ocean we attributed the value of “no data”. The landscape disturbance index of each 100×100 m cell in the island was then calculated as:
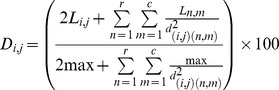
where: *D_i,j_* is the final index value of the cell in row i and column j; *L* is the local disturbance value of each cell (as defined above); *r* is number of rows in the map; *c* is number of columns in the map; *d* is the distance between the centroids of each two cells; *max* is the maximum theoretical value of disturbance each cell may take (in this case max = 6, corresponding to urban/industrial areas). Thus, the influence of each cell surrounding the focal cell is inversely proportional to the square of the distance between the two cells. That is, a cell next to the focal cell (*d* = 1) has 4 times more influence that a cell two rows apart (*d* = 2). Although all cells in the island were taken into account, the ones far away from the focal cell had an almost negligible individual influence. The *L* value of the focal cell was multiplied by 2 to guarantee that the land use of the focal cell (*d* = 0) is double weighted compared with the immediately surrounding cells (*d* = 1). Also, the division by the maximum value of each cell was necessary to guarantee that the presence of a land use without L values at the border of the study area (in our case the ocean) would not make all border (coastal) cells having low *D* (as if the ocean was equivalent to natural forest (*L* = 0)). As a beneficial side-effect, this division by the theoretical maximum also guarantees that *D* is not open-scaled, since *D* is limited between 0 (no disturbance at all, only possible if all cells in the island had natural forest) and 100 (maximum possible disturbance, only possible if all cells in the island were occupied by urban/industrial areas).

### Identifying species response to disturbance

To identify species response to disturbance, we use Threshold Indicator Taxa ANalysis (TITAN). TITAN is a recently developed method [18] allowing the identification of threshold(s) or change point(s) along environmental gradients for each taxon. Basically, TITAN detects changes in species distributions along an environmental gradient over space or time, and assesses synchrony among species change points as evidence for community thresholds. TITAN uses IndVal (Indicator Value) scores [39] to identify change points along a continuous environmental gradient. Indval is a method based on the comparison of relative abundances and relative frequencies of species in different groups of sites chosen a priori. A good indicator species is one which occurs at all sites in a given group and never in any other groups [39]. Here, midpoints between observed values of disturbance are candidate change points (x_i_) used to iteratively split observations into two groups, and thus produce two IndVal scores at each split. The relative magnitude of IndVal scores for groups on each side of a candidate change point reflects whether a species shows greater association with the left (negative response with respect to x = associated with low disturbance) or the right (positive response = associated with high disturbance) side of each split. The greater the difference in association created by a specific split, the greater the IndVal score for one of the two groups (i.e. low or high disturbance). The greatest IndVal score at each split and the direction of the split (i.e. toward low disturbance or toward high disturbance) on which it occurs are kept for comparison with those at other candidate change points. The probability of obtaining an equal or larger IndVal score from random data is estimated by comparing the magnitude of each observed IndVal score with those generated by randomly assigning group membership via permutations [39]. TITAN uses bootstraps to compute the confidence interval of the change point location along the gradient for each taxon. Additionally, this bootstrap procedure is also used to estimate two new measures for each taxon: the “purity” (proportion of the bootstrap replicates with the same response direction, i.e., negative or positive), and “reliability” (proportion of the replicates with *p*-values for the indicator value score at the change point below a specified probability). Therefore, a species may be considered significantly associated to either low or high disturbance if IndVal <0.05, purity >0.95 and reliability >0.95.

### Identifying community disturbance threshold

By using the disturbance thresholds identified for each species (see section above), subsequently TITAN is also able to identify two community thresholds both associated with low and high disturbance. To estimate these two thresholds, the IndVal scores are first standardized to *z* scores (i.e. by subtracting the mean of the randomization from the observed IndVal and dividing by the SD of the randomization) to describe the magnitude of the response relative to each species' abundance distribution. Standardization is required to allow rare species with small indicator value scores to have high z scores if they show a sharp response at a particular point along the disturbance gradient. Then, species are grouped according to the direction of the response into *z−* species, with a negative response (low disturbance affinity), and *z+* species, with a positive response (high disturbance affinity), along the disturbance gradient. Finally, the *z−* and *z+* values at each point along the gradient are summed. Then, negative and positive community thresholds correspond to the disturbance value where the sum (*z−*) or sum (*z+*) scores show a peak, respectively. These two community thresholds can be subsequently mapped to visually identify which sectors of the study areas (in our case, the study island) they delimited.

### Investigating relationship between species response and species attributes

The TITAN analysis distinguishes two species groups: (1) species significantly associated with low disturbance (i.e. negative z− response; significant IndVal scores <0.05, purity >0.95 and reliability >0.95), and (2) species significantly associated to high disturbance (i.e. positive z+ response; significant IndVal scores <0.05, purity >0.95 and reliability >0.95). We therefore used TITAN results to test for potential association between species attributes and species response to disturbance. We considered four main characteristics: biogeographic categories (endemics, natives non-endemics and exotics), taxonomic order, feeding guild (predator, herbivore, fungivore and saprophagous) and body size. Since our data were greatly unbalanced (i.e. sometimes one species per characteristics' level), we tested each factor independently by using a series of χ^2^ tests and non-parametric tests. First, we tested whether biogeographic categories, taxonomic orders, feeding guilds or body sizes differed between the two groups of species (i.e. between species with negative response and species with positive response). χ^2^ tests were performed to assess differences in species frequency of the first three characteristics, while differences in body size were assessed by performing Kruskal-Wallis tests. Second, we assessed whether taxonomic order, feeding guild or body size differed among biogeographic categories within each group of species by using χ^2^ tests and Kruskal-Wallis tests. To account for potential expected frequencies below 5, all p-values for the χ^2^ tests were computed by permutations tests using Monte Carlo simulations. For Kruskal-Wallis, if significant differences were detected, pairwise Mann Whitney-U post hoc tests were implemented.

### Statistical analysis software

All the analyses were performed using R [40]. Particularly, we used the R code provided by Baker & King [18] to implement TITAN in R 2.13.0. Abundance data were transformed (log_10_(x+1)) to reduce the influence of highly abundant species on IndVal scores [18]. Following the recommendations of Baker & King [18], taxa occurring in less than 3 sites across the gradient were deleted to remove outliers representing a potential bias. In our study, we also use a minimum group size of five observations to compute IndVal for TITAN analyses. We reran TITAN across 500 bootstrap replicates to compute purity and reliability of individual threshold indicator taxa and uncertainty surrounding thresholds based on the distribution of maximum TITAN sum(z−) (individual taxa) and TITAN sum (z+) values.

## Results

**Figure 1:**
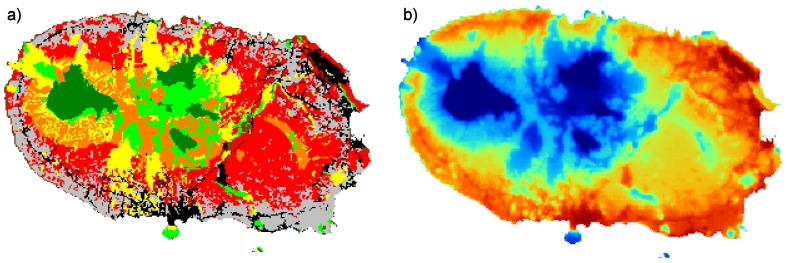
Maps of Terceira Island with (a) distribution of land use types and (b) value of landscape disturbance. For land use types (a): dark green = natural forests, light green = natural(ized) vegetation, yellow = exotic forests, orange = semi-natural pastures, red = intensively managed pastures, grey = orchards/agriculture areas, black = urban/industrial areas and brown = rocky outcrops). For landscape disturbance gradient (b): values of landscape disturbance are represented in a gradient from blue for lowest values to red for highest values.

The landscape disturbance index was calculated for all the 100×100 m cells in Terceira Island and mapped (Fig. 1). Although it closely follows the distribution of different land uses, it allowed obtaining a continuous landscape of disturbance values for the entire island. For the 72 sites sampled across the different land uses, the index ranged from D = 14.38 (the lowest disturbed site) to D = 75.58 (the highest disturbed site).

**Figure 2:**
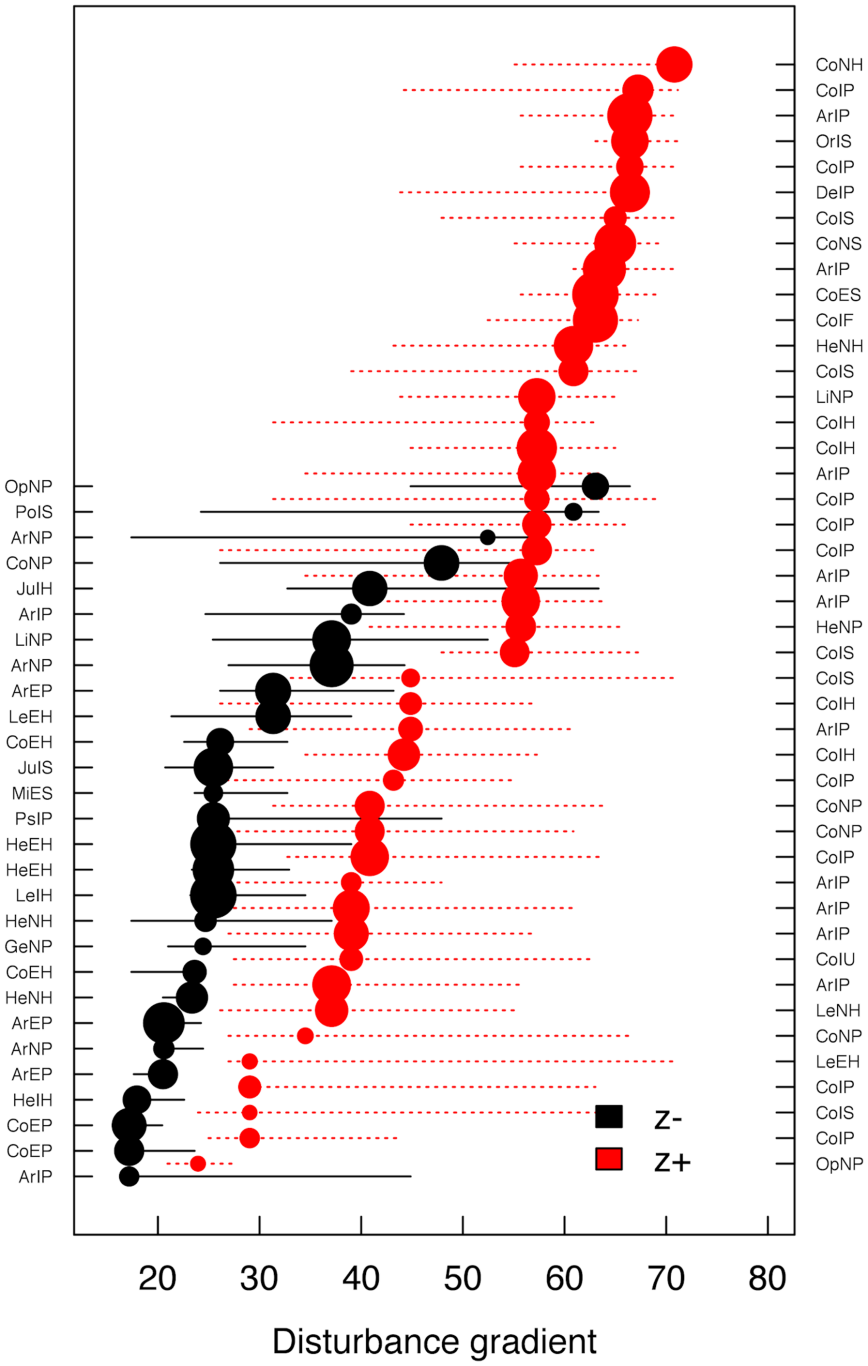
Change points and 90% confidence limits of significant indicator species (n = 72) along the disturbance gradient. Significant indicator species are species with, IndVal p < 0.05, purity > 0.95 and reliability > 0.95 for 500 bootstrap and 250 permutation replicates. Change points are represented by black and red circles for species associated with low and high disturbance respectively and are sized in proportion to the magnitude of the response (z scores; see Materials and methods). Code for species on vertical axis (both left and right): The two first letters: Order (e.g. Ar = Araneae, He = Hemiptera, etc.; see Supporting Information S1); the third letter is the biogeographic category (E = endemic, N = Native non-endemic and I = Introduced/Exotic). The last letter is the feeding guild (P = predator, H = herbivore, F = Fungivore, S = Saprophagous and U = Undetermined).

The TITAN framework identified 72 species out of the total 140 analysed (51%) with significant indicator value for disturbance (Supporting Information S1). Twenty-eight species composed the group significantly associated with low disturbed sites (i.e. corresponding to 38% of the 72 species identified). The disturbance threshold of these species ranged from 17.17 to 60.17 but 24 of the 28 species had a threshold below 40 (Fig. 2). Forty-four species composed the group associated with high disturbance (i.e. corresponding to 62% of the 72 species identified) with a threshold ranging from 23.94 to 70.80.

**Figure 3:**
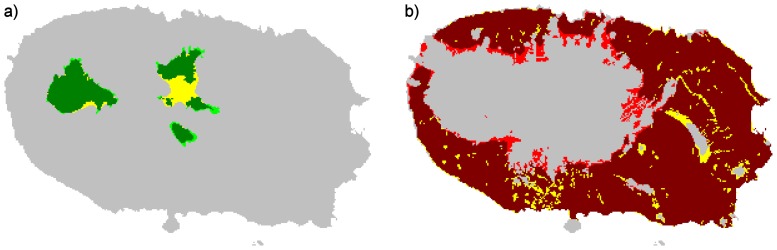
Maps of Terceira Island with (a) areas below the community threshold for the negative response (sum *z−*) and (b) areas above the community thresholds for the positive response (sum *z+*). (a) Areas below the community thresholds for the negative responses (sum *z−*) of species to disturbance gradient are native forests (dark green) and other habitats (yellow). Areas of native forest that are above this threshold are also represented (light green). (b) Area above the community thresholds for the positive (sum *z+*) responses of species to disturbance gradient are the intensively managed pastures, orchards/agriculture areas or urban/industrial areas (dark red) and other habitats (yellow). Intensively managed pastures, orchards/agriculture areas or urban/industrial areas that are below this threshold are also represented (light red).

Additionally, the TITAN analysis allowed estimating two community-level-thresholds associated respectively with low and high disturbance by identifying peaks in distribution of the sum (z−) and sum (z+) along the gradient (Table 1). The distribution of both sum (z−) and sum (z+) did not show abrupt peaks meaning that the existence of clear disturbance thresholds is not evident. Therefore, these thresholds should be interpreted with caution. This uncertainty is also reflected in the large confidence limits associated with them (Table 1). The threshold associated with low disturbance roughly followed the distribution of native forests (Fig. 3a), although other habitats connecting some of the forest fragments were also included and many forest margins were excluded. The threshold associated with high disturbance roughly followed the distribution of intensively managed pastures, orchard/agriculture areas and urban/industrial areas (Fig. 3b), although marginal areas were either included or excluded depending on the surrounding landscape.

**Table 1:** Community-thresholds and its associated percentiles.

	Thresholds	0.05	0.10	0.50	0.90	0.95
sum *z−*	25.458	21.683	23.194	25.458	32.734	37.091
sum *z+*	57.274	25.458	25.458	55.092	63.921	64.985

Community-thresholds are based on the sum of the *z−* and *z+*. Associated percentiles correspond to the frequency distribution of thresholds from 500 bootstrap replicates.

**Figure 4:**
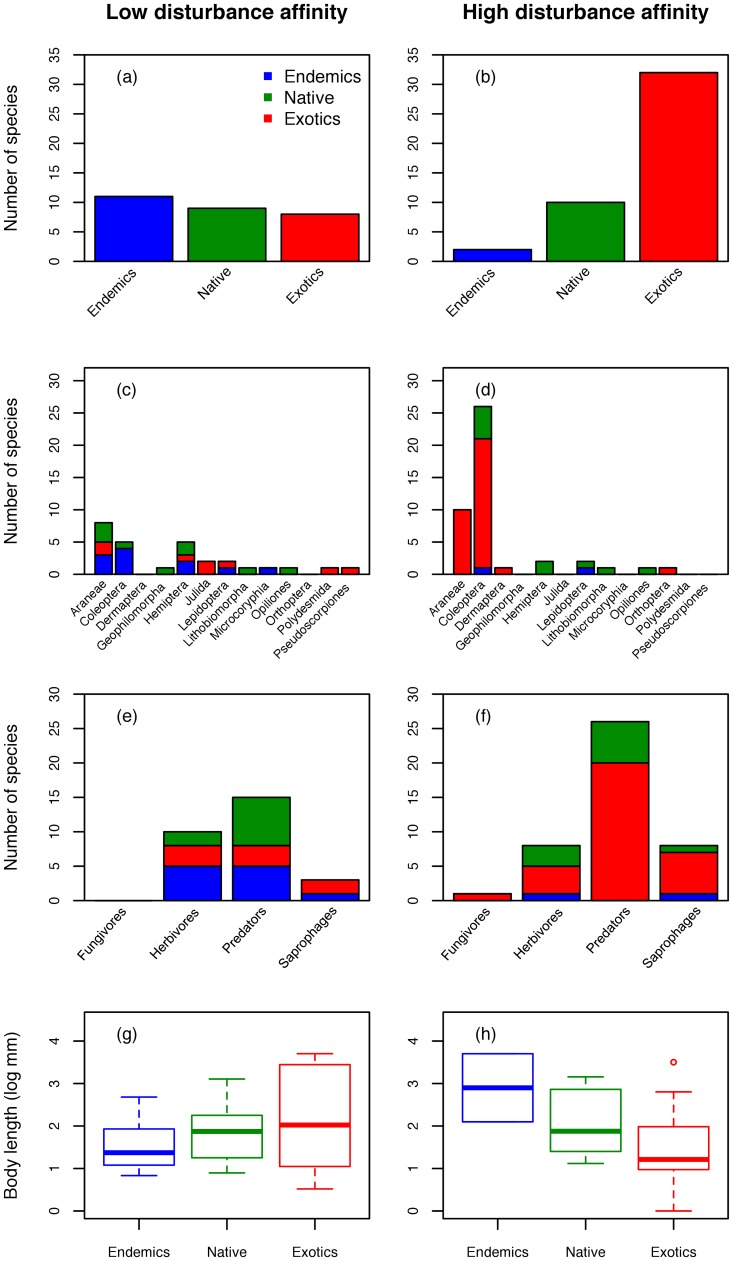
Composition in biogeographic categories (a-b), taxonomic order (c-d), feeding guild (e-f) and body size (g-h) for the species group associated with low and high disturbance (n = 28 and n = 44 respectively). The three biogeographic categories (endemics, natives and exotics) are also distinguished in the panels c to h for correspondence with the contingency table and body size distributions used in within groups tests (See table 1). Note that for the species group associated with low disturbance, 43 species out of the 44 were considered since feeding guild was undetermined for one species (see Supporting Information S1). Boxplots indicate the distribution of the body size per biogeographic category. For graphical convenience, body size was log-transformed.

Investigation of the link between species response groups and species characteristics revealed significant differences in biogeographic categories and taxonomic order composition (Table 2; Fig. 4). As expected, most of the endemic species are significantly associated with low disturbance areas and most of the exotic species are significantly associated with high disturbance areas. However, within the group of species associated with low disturbance, the three biogeographic categories are almost equally represented with 11 endemic, 9 native and interestingly, 8 exotic species. Exotics clearly dominate the group associated with high disturbance areas with 32 exotic, 10 native and only two endemic species. Species associated with low disturbance are taxonomically diverse, but Araneae (n = 8), Coleoptera (n = 5) and Hemiptera (n = 5) are the most species rich. While the Coleoptera are almost all endemics, there are a few native and even exotic spiders and Hemiptera associated with low disturbed sites. Among the species associated with high disturbance, Coleoptera (n = 26) and, in a lesser extent, Araneae (n = 10), dominate. All spiders and most beetles are exotics. Feeding guild and body size did not show any significant association with species response groups. Within the group associated with low disturbance, no significant differences were found between endemics, natives and exotics in their taxonomic order composition, feeding guild or body size. Within the group associated with high disturbance, only body size was slightly different between the three biogeographic categories. However, the Mann Whitney-U post hoc tests did not show significant pairwise comparisons (All p>0.05).

**Table 2:** Results of the χ^2^ and Kruskal-Wallis tests performed between and within the two species groups identified by TITAN analysis.

	Characteristic	*χ^2^*	*p*
Between groups			
	biogeographic categories	**18.017**	**<0.001**
	Taxonomic order	**19.722**	**0.006**
	Feeding guild	3.430	0.330
	Body size	0.028	0.867
Between colonization categories within groups			
Negative response			
	Taxonomic order	23.008	0.249
	Feeding guild	4.778	0.317
	Body size	1.063	0.587
Positive response			
	Taxonomic order	29.530	0.072
	Feeding guild	5.447	0.452
	Body size	**8.302**	**0.015**

Between groups, difference in taxonomic compositions, feeding guild and biogeographic categories and body size were assessed independently. Within groups, difference in taxonomic compositions, feeding guild and body size were assessed between biogeographic categories. The χ^2^ values and its associated p.value were given for both χ^2^ test and Kruskal-Wallis since the Kruskal-Wallis statistic is a very close approximation of the chi-square distribution. For χ^2^ tests, p.value were computed by permutations tests using Monte Carlo simulations. Significant results are marked in bold.

## Discussion

The general goal of this study was to illustrate a new approach for spatial analysis in conservation biology. This was based on a two-step protocol: (1) the creation of a new metric that provides a continuous measure of disturbance for any focal community on the basis of the surrounding landscape matrix (disturbance index *D*) and; (2) the use of a recently developed analysis of taxa responses to specific gradients to estimate species and community-level disturbance thresholds (TITAN analysis). Spatial detection of anthropogenic disturbance effects on species communities is a cornerstone concern in conservation biology and we believe that the approach proposed here may be useful for future researches.

### Disturbance index

Ecologists often consider disturbance levels as being well represented by habitat categories (e.g. native vs. anthropogenic habitats [41], [42]). Although this approach is useful in many contexts and often the only possible option due to the lack of data, disturbance is more likely to be a continuous phenomenon than a discrete one [43]. A number of metrics have been previously developed to measure disturbance on a continuous scale. These may be based on multiple factors that are thought to disrupt community composition and may be intrinsic to the focal sites, such as urbanization, logging, trampling, livestock grazing and land degradation or dependent on the surrounding landscape matrix, such as fragmentation or connectivity between patches [44]–[47]. All these may be studied either in isolation or through aggregated indices. Taking the surrounding landscape into account is, however, critical, given the meta-population and source-sink dynamics that necessarily occur among different habitats [32], [48], which may determine that habitat fragments with similar local disturbance levels may present different species compositions depending on the surrounding matrix [45], [49]. In a previous work on Terceira Island, Borges et al. [32], [48] found that a source-sink dynamics between different habitats was, in fact, critical to understand community composition, particularly in marginal areas of forest fragments. Our newly developed disturbance measure reflects the effects of both local and regional processes in a single, continuous metric. Consequently, it can be viewed as a simple univariate gradient, easy to integrate in many statistical frameworks and with a wide application in conservation biology studies.

Transforming a simple ordinal scale of disturbance intensity into a continuous measure depicts disturbance patterns in a more realistic way, especially in buffer zones between different land uses. In our application of the new index D, L-values of local disturbance were arbitrarily established by the authors according to the knowledge of the studied system and the aim of the study. In general, L values can be determined a priori to reflect environmental disturbance, independently of the characteristics of the group of concern as done here. For this, the researcher identifies a number of basic land use types or habitats that reflect different levels of anthropogenic impact and assigns a score to each category [50]. For example, completely artificial habitats, such as urban or industrial areas, are assumed to be the most disturbed, thus receiving the highest score, whereas virtually pristine habitats, such as primary forests, receive the lowest score. What constitutes the extremes of the scale will depend on the particular context. For example, in certain ecosystems, such as most areas in Europe, where even well-preserved biotopes suffered some kind of disturbance starting in prehistoric times, the currently ‘most natural’ settings are always the result of a certain disturbance, thus the lowest score will be given to habitats that are in fact not pristine. Regarding the scale to adopt, the most obvious solution is a linear one, and this can be particularly appropriate if transitions among habitats are assumed to be sufficiently smooth, as in our case study. However, other scales, such as a geometric series, might be appropriate if one would want the values to reflect strongest differences between natural or semi-natural habitats vs. highly impacted or completely artificial habitats. When L values are intended to express ‘absolute’ disturbance, the index can be used to identify which species respond negatively, and which respond positively, to increasing disturbance, and this is the approach illustrated in the case study discussed here.

We can also imagine a reverse situation, in which the researcher uses a particular indicator group to evaluate the disturbance level of the areas or habitat types. In this case, the response of the indicator group to disturbance can be used to assign the scores to the habitat/land use types. For example, species known to increase their population densities with increasing disturbance (as commonly found for invasive plants or opportunistic animals, such as many birds associated with urban areas) can be used to assign scores to habitats according to their frequencies or abundances in each habitat.

Besides integrating both local and regional processes and being a continuous measure, the index we propose has the additional advantage that it is particularly adequate for mapping disturbance of large regions, as it does not require measuring a number of variables at all single sites/cells/habitat patches in a region of interest. This allows, among other applications, the use of this metric to model predicted species distributions [51] accounting for the disturbance value of each site/cell. In fact, we used our disturbance index here presented to model 47 arthropod species in Terceira Island and it had a high explanatory power for the distribution of most species, being one of the main contributing variables to most final models [52].

### Indicator species analysis

As explanatory variables, such as diverse disturbance sources, may be studied in isolation or in combined metrics, response variables, usually the presence/absence or the abundance of taxa, may also be studied either in isolation or as a combined community metric. Using the newly developed TITAN protocol [18] allows both options under the same framework. This analysis allowed us to simultaneously look for individual indicator species and analyse their characteristics and discover community thresholds common to most of the species.

As previously suggested [26], local composition of arthropod communities in Terceira is mostly determined by the presence of nearby natural forests or intensively managed pastures. To the latter land use we may add agricultural and urban/industrial areas. Native forests are the main source for endemic species, whereas intensively managed pastures and even more highly disturbed land uses are the main source for exotic species [26], [32]. Therefore, their borders are found by the TITAN analysis as community thresholds for species responding negatively and positively to disturbance respectively. Native non-endemic species as a group do not show clear patterns, as although they are naturally occurring in the islands, they tend to be less specialized in habitat type. In fact, the Azorean Laurissilva is a very particular forest, completely different from, for example, the Madeira Laurissilva, which has both a different plant species composition and a radically different structure [53]. Forest trees in Madeira present a high stature and the ground is covered by leaf litter, whereas the trees in the Azores are contrastingly low in stature and the ground is mostly covered by mosses and ferns. Native species, which occur in other regions besides the Azores, must therefore be able to cope with different habitats, and this is evident from our results. The communities present in semi-natural pastures and exotic forests are a mixture of the other land uses, although they seem to play an important role as corridors between natural forests for both endemic and native species or even as a refuge for some endemic species [26], [30].

Analyzing the two orders with most indicator species, spiders and beetles, the differences are particularly evident. Spiders identified as indicators of low disturbance sites are mostly endemic or native although two exotic species, *Agyneta decora* and *Ero furcata* mostly occur in native forests. It is hard to know the impact of these species on the native and especially the endemic assemblages because closely related and therefore competing species may have been driven to extinction already, either in Terceira Island only or in the archipelago [29]. The first species has a close endemic relative in the Azores, *Agyneta rugosa*. Interestingly, although both species occupy different islands from West to East of the archipelago, they only co-occur on São Jorge, an island that, while presenting few and small native forest patches, has some of the most undisturbed forests in the archipelago, namely Topo, which is a small fragment with two single island endemic spider species [44], [52], [54]. *Ero furcata* is a specialist spider-hunter, deceiving web-builders in their own snares as most members of the family Mimetidae [55]. As no other araneophagic spiders live in the archipelago except for three exotic *Ero*, these species may have just occupied an empty niche. Their impact on their spider prey is however unknown. All spiders species identified as indicators of disturbed sites are exotic species, reflecting the lack of particular adaptation of native and endemic species to such habitats.

Beetles indicators of low disturbance sites are mostly endemic, and include species known previously to be dependent of high altitude pristine native forests [48], [56]. Many endemic species only occur in native core areas, distant to the nearest ecotone, confirming previous findings [48]. On the contrary, generalist species experiencing landscape source-sink dynamics can cope with local disturbance in a dynamic fragmented landscape [57]. One endemic beetle, *Heteroderes azoricus*, is indicator of highly disturbed habitats, a fact also demonstrated in previous studies [30].

It should be noted, however, that the TITAN approach uses a step-function model of community threshold, one which tries to identify steep changes in the frequency or abundance of taxa along environmental gradients. In many cases the change may be gradual, such as in broken-stick or dose-response models [58], [59]. In such cases great care should be taken in the interpretation of results. In our study, the community thresholds were correctly identified, as the disturbance index is based on the spatial distribution of discrete land use types. Moreover, results corroborate previous studies [26] and match our empirical knowledge of the ecology of the studied communities and individual species.

## Conclusions

Concluding, the landscape disturbance index coupled with the TITAN analysis allows perceiving patterns that would probably go unnoticed otherwise. This framework may be useful in many different situations, namely: (1) when both local and regional processes are to be reflected on single disturbance measures; (2) when these are better quantified in a continuous gradient; (3) when mapping disturbance of large regions using fine scales is necessary; (4) when indicator species for disturbance are searched for and; (5) when community thresholds are useful to understand the global dynamics of habitats.

## Supporting Information

Supporting Information S1 **List of the 72 species having significant response with either low or high disturbance and their corresponding threshold (with associated 90% confidence limits) and the** ***z*** **-score (See material and methods).** Taxonomic information as well as biogeographic categories, feeding guild and body size are also given.
(PDF)

## References

[1] Malanson GP, Cramer BE (1999) Landscape heterogeneity, connectivity, and critical landscapes for conservation. Divers Distrib 5: 27–40.

[2] Ricketts TH (2001) The matrix matters: effective isolation in fragmented landscapes. Am Nat 158: 87–99. 18707317 10.1086/320863

[3] Fischer J, Lindenmayer D (2007) Landscape modification and habitat fragmentation: a synthesis. Global Ecol Biogeogr 16: 265–280.

[4] Didham RK, Hammond PM, Lawton JH, Eggleton P, Stork NE (1998) Beetle species responses to tropical forest fragmentation. Ecol Monogr 68: 295–323.

[5] Duelli P, Marchand M, Studer I, Jakob S (1990) Population movements of arthropods between natural and cultivated areas. Biol Conserv 54: 193–207.

[6] Peèr G, van Maanen C, Turbé A, Matsinos YG, Kark S (2011) Butterfly diversity at the ecotones between agricultural and semi natural habitats across a climatic gradient. Divers Distrib 17: 1186–1197.

[7] Pryke JS, Samways MJ (2012) Conservation of complex natural forest and plantation edge effects. Landscape Ecol 27: 73–85.

[8] Ries L, Fletcher RJJ, Battin J, Sisk TD (2004) Ecological responses to habitat edges: mechanisms, models and variability explained. Annu Rev Ecol Evol Syst 35: 491–522.

[9] Toms J, Lesperance ML (2003) Piecewise regression: a tool for identifying ecological thresholds. Ecology 84: 2034–2041.

[10] Huggett AJ (2005) The concept and utility of ‘ecological thresholds’ in biodiversity conservation. Biol Conserv 124: 301–310.

[11] Groffman PM, Baron JS, Blett T, Gold AJ, Goodman I, et al (2006) Ecological thresholds: the key to successful environmental management or an important concept with no practical application? Ecosystems 9: 1–13.

[12] Donaldson J, Nänni I, Zachariades C, Kemper J (2002) Effects of habitat fragmentation on pollinator diversity and plant reproductive success in renosterveld shrublands of South Africa. Conserv Biol 16: 1267–1276.

[13] Ewers RM, Didham RK (2006) Continuous response functions for quantifying the strength of edge effects. J Appl Ecol 43: 527–536.

[14] Brenden TO, Wang L, Su Z (2008) Quantitative identification of disturbance thresholds in support of aquatic resource management. Environ Manage 42: 821–832. 18491181 10.1007/s00267-008-9150-2

[15] Andersen T, Carstensen J, Hernandez-Garcia E, Duarte CM (2009) Ecological regime shifts: approaches to identification. Trends Ecol Evol 24: 49–57. 18952317 10.1016/j.tree.2008.07.014

[16] Sonderegger DL, Wang H, Clements WH, Noon BR (2009) Using SiZer to detect thresholds in ecological data. Front Ecol Environ 7: : 190–195.

[17] Starzomski BM, Srivastava DS (2007) Landscape geometry determines community response to disturbance. Oikos 116: 690–699.

[18] Baker ME, King RS (2010) A new method for detecting and interpreting biodiversity and ecological community thresholds. Methods Ecol Evol 1: 25–37.

[19] King RS, Baker ME (2011) An alternative view of ecological community thresholds and appropriate analyses for their detection: comment. Ecol Appl 21: 2833–2839. 22073663 10.1890/10-0882.1

[20] Whittaker RJ, Fernández-Palacios JM (2007) Island biogeography: ecology, evolution, and conservation, 2nd edition. Oxford: Oxford University Press.

[21] Jäger H, Tye A, Kowarik I (2007) Tree invasion in naturally treeless environments: impacts of quinine (Cinchona pubescens) trees on native vegetation in Galápagos. Biol Conserv 140: 297–307.

[22] Drake DR, Hunt TL (2009) Invasive rodents on islands: integrating historical and contemporary ecology. Biol Invasions 11: 1483–1487.

[23] Caujape-Castells J, Tye A, Crawford DJ, Santos-Guerra A, Sakai A, et al (2010) Conservation of oceanic island floras: present and future global challenges. Perspect Plant Ecol Evol Syst 12: 107–129.

[24] Paulay G (1994) Biodiversity on oceanic islands: its origin and extinction. Am Zool 34: 134–144.

[25] Steadman DW (2006) Extinction and biogeography of tropical pacific birds. London: University of Chicago Press.

[26] Cardoso P, Aranda SC, Lobo JM, Dinis F, Gaspar C, et al (2009) A spatial scale assessment of habitat effects on arthropod communities of an oceanic island. Acta Oecol 35: 590–597.

[27] Triantis KA, Borges PAV, Ladle RJ, Hortal J, Cardoso P, et al (2010) Extinction debt on oceanic islands. Ecography 33: 285–294.

[28] Connor SE, van Leeuwen JFN, Rittenour TM, van der Knaap WO, Ammann B, et al (2012) The ecological impact of oceanic island colonization – a palaeoecological perspective from the Azores. J Biogeo 39: 1007–1023.

[29] Cardoso P, Arnedo MA, Triantis KA, Borges PAV (2010) Drivers of diversity in Macaronesian spiders and the role of species extinctions. J Biogeo 37: 1034–1046.

[30] Meijer SS, Whittaker RJ, Borges PAV (2011) The effects of land-use change on arthropod richness and abundance on Santa Maria Island (Azores): unmanaged plantations favour endemic beetles. J Insect Conserv 15: 505–522.

[31] Borges PAV, Brown VK (2004) Arthropod community structure in pastures of an island archipelago (Azores): looking for local-regional species richness patterns at small-scales. Bull Entomol Res 94: 111–121. 15153294 10.1079/ber2004289

[32] Borges PAV, Ugland KI, Dinis FO, Gaspar C (2008) Insect and spider rarity in an oceanic island (Terceira, Azores): true rare and pseudo-rare species. In: Fattorini S, editor. Insect Ecology and Conservation. Kerala: Research Signpost. pp. 47–70.

[33] Carvalho JC, Cardoso P, Borges PAV, Schmera D, Podani J (2013) Measuring fractions of beta diversity and their relationships to nestedness: a theoretical and empirical comparison of novel approaches. Oikos in press.

[34] Borges PAV, Costa A, Cunha R, Gabriel R, Gonçalves V, et al. (2010) Description of the terrestrial and marine biodiversity of the Azores. In: Borges PAV, Costa A, Cunha R, Gabriel R, Gonçalves V, et al.., editors. A list of the terrestrial and marine biota from the Azores. Oeiras: Princípia. pp. 9–33.

[35] Cardoso P, Erwin TL, Borges PAV, New TR (2011) The seven impediments in invertebrate conservation and how to overcome them. Biol Conserv 144: 2647–2655.

[36] Borges PAV, Gaspar CS, Santos AMC, Ribeiro SP, Cardoso P, et al.. (2011) Patterns of colonization and species distribution for Azorean arthropods: evolution, diversity, rarity and extinction. In: Martins AMF, Carvalho MC, editors. Celebrating Darwin: Proceedings of the Symposium “Darwin's Mistake and what we are doing to correct it”, Ponta Delgada, 19–22 September, 2009. Açoreana. pp. 93–123.

[37] Moran VC, Southwood TRE (1982) The guild composition of arthropod communities on trees. J Anim Ecol 51: 289–306.

[38] DROTRH (2008) Carta de ocupação do solo da região Autónoma dos Açores - Projecto SUEMAC. Ponta Delgada: Secretaria Regional do Ambiente, Direcção Regional do Ordenamento do território e dos Recursos Hídricos.

[39] Dufrêne M, Legendre P (1997) Species assemblages and indicator species: the need for a flexible asymmetrical approach. Ecol Monogr 67: 345–366.

[40] R Development Core Team (2009) R: a language and environment for statistical computing. Vienna: R Foundation for Statistical Computing. Available: http://www.R-project.org.

[41] Lawton JH, Bignell DE, Bolton B, Bloemers GF, Eggleton P, et al (1998) Biodiversity inventories, indicator taxa and effects of habitat modification in tropical forest. Nature 391: 72–76.

[42] Basset Y, Missa O, Alonso A, Miller SE, Curletti G, et al (2008) Choice of metrics for studying arthropod responses to habitat disturbance: one example from Gabon. Insect Conserv Diver 1: 55–66.

[43] Turner MG (2010) Disturbance and landscape dynamics in a changing world. Ecology 91: 2833–2849. 21058545 10.1890/10-0097.1

[44] Cardoso P, Borges PAV, Gaspar C (2007) Biotic integrity of the arthropod communities in the natural forests of Azores. Biodivers Conserv 16: 2883–2901.

[45] Bonte D, Maes D (2008) Trampling affects the distribution of specialised coastal dune arthropods. Basic Appl Ecol 9: 726–734.

[46] Comor V, Orgeas J, Ponel P, Rolando C, Delettre Y (2008) Impact of anthropogenic disturbances on beetle communities of French Mediterranean coastal dunes. Biodivers Conserv 17: 1837–1852.

[47] Martorell C, Peters EM (2009) Disturbance-response analysis: a method for rapid assessment of the threat to species in disturbed areas. Conserv Biol 23: 377–387. 19183208 10.1111/j.1523-1739.2008.01134.x

[48] Borges PAV, Lobo JM, Azevedo EB, Gaspar C, Melo C, et al (2006) Invasibility and species richness of island endemic arthropods: a general model of endemic vs. exotic species. J Biogeo 33: 169–187.

[49] Kallimanis AS, Kunin WE, Halley JM, Sgardelis SP (2005) Metapopulation extinction risk under spatially autocorrelated disturbance. Conserv Biol 19: 534–546.

[50] Laliberté E, Wells JA, DeClerck F, Metcalfe DJ, Catterall CP, et al (2010) Land-use intensification reduces functional redundancy and response diversity in plant communities. Ecol Lett 13: 76–86. 19917052 10.1111/j.1461-0248.2009.01403.x

[51] Elith J, Graham CH, Anderson RP, Dudik M, Ferrier S, et al (2006) Novel methods improve prediction of species' distributions from occurrence data. Ecography 29: 129–151.

[52] Fattorini S, Cardoso P, Rigal P, Borges PAV (2012) Use of arthropod rarity for area prioritisation: insights from the Azorean islands. PLoS One 7: e33995. 22479498 10.1371/journal.pone.0033995 PMC3316514

[53] Jardim R, Menezes de Sequeira M (2008) The vascular plants (Pteridophyta and Spermatophyta) of the Madeira and Selvagens archipelagos. A list of the terrestrial fungi, flora and fauna of Madeira and Selvagens archipelagos. In: Borges PAV, Abreu C, Aguiar AMF, Carvalho P, Jardim R, et al.., editors. Funchal and Angra do Heroísmo: Direcção Regional do Ambiente da Madeira and Universidade dos Açores. pp. 157–207.

[54] Gaspar C, Gaston KJ, Borges PAV, Cardoso P (2011) Selection of priority areas for arthropod conservation in the Azores archipelago. J Insect Conserv 15: 671–684.

[55] Cardoso P, Pekár S, Jocqué R, Coddington JA (2011) Global patterns of guild composition and functional diversity of spiders. PLoS One 6: e21710. 21738772 10.1371/journal.pone.0021710 PMC3126856

[56] Gaston KJ, Borges PAV, He F, Gaspar C (2006) Abundance, spatial variance and occupancy: arthropod species distribution in the Azores. J Anim Ecol 75: 646–656. 16689947 10.1111/j.1365-2656.2006.01085.x

[57] Tscharntke T, Klein AM, Kruess A, Dewenter IS, Thies C (2005) Landscape perspectives on agricultural intensification and biodiversity-ecosystem service management. Ecol Lett 8: 857–874.

[58] Cuffney TF, Brightbill RA, May JT, Waite IR (2010) Responses of benthic macroinvertebrates to environmental changes associated with urbanization in nine metropolitan areas of the conterminous United States. Ecol Appl 20: 1384–1401. 20666256 10.1890/08-1311.1

[59] Cuffney TF, Qian SS, Brightbill RA, May JT, Waite IR (2011) Response to King and Baker: limitations on threshold detection and characterization of community thresholds. Ecol Appl 21: 2840–2845.

