# Supplementary material for: Integrating Landscape Disturbance and Indicator Species in Conservation Studies
Source: PLoS One. 2013 May 1;8(5):e63294. doi: 10.1371/journal.pone.0063294 (PMC3641138; doi:10.1371/journal.pone.0063294)
Supplement: Supporting Information S1 — List of the 72 species having significant response with either low or high disturbance and their corresponding threshold (with associated 90% confidence limits) and the z -score (See material and methods). Taxonomic information as well as biogeographic categories, feeding guild and body size are also given. (PDF) [file pone.0063294.s001.pdf]

Supplementary Information S1. List of the 72 species having significant response with either low or high disturbance and their corresponding threshold (with associated 90% confidence limits) and the z-score (See material and methods). Taxonomic information as well as biogeographic categories (Geo.; End = Endemic, Nat = Native non-endemic, Exo = Exotics), feeding guild (Feed.; P = Predator, H = Herbivore, F = Fungivore, S = Saprophagous, U = Undetermined) and body size (BS) are also given.

code	Class	Order	Family	Genus	Species	Threshold	CL 0.05	CL 0.95	z score	Resp.	Geo.	Feed.	BS
ArIP	Arachnida	Araneae	Linyphiidae	*Agyneta*	*decora*	17.178	17.178	47.901	4.88	Low	EXO	P	2.75
ArEP	Arachnida	Araneae	Linyphiidae	*Lepthyphantes*	*acoreensis*	31.34	24.982	44.197	9.64	Low	END	P	3.25
ArNP	Arachnida	Araneae	Linyphiidae	*Palliduphantes*	*schmitzi*	52.443	17.178	60.879	3.46	Low	NAT	P	1.95
ArEP	Arachnida	Araneae	Linyphiidae	*Porrhomma*	*borgesi*	20.501	17.395	21.948	7.85	Low	END	P	2
ArNP	Arachnida	Araneae	Linyphiidae	*Tenuiphantes*	*miguelensis*	37.092	25.459	47.901	12.04	Low	NAT	P	2.65
ArIP	Arachnida	Araneae	Mimetidae	*Ero*	*furcata*	39.022	21.077	47.901	5.11	Low	EXO	P	2.88
ArEP	Arachnida	Araneae	Theridiidae	*Rugathodes*	*acoreensis*	20.592	20.526	24.687	11.33	Low	END	P	1.8
ArNP	Arachnida	Araneae	Thomisidae	*Xysticus*	*cor*	20.592	20.501	24.687	5.2	Low	NAT	P	7
OpNP	Arachnida	Opiliones	Phalangiidae	*Leiobunum*	*blackwalli*	63.037	43.174	67.323	6.98	Low	NAT	P	6
PsIP	Arachnida	Pseudoscorpiones	Chthoniidae	*Chthonius*	*ischnocheles*	25.459	23.615	55.693	8.75	Low	EXO	P	2
GeNP	Chilopoda	Geophilomorpha	Geophilidae	*Geophilus*	*truncorum*	24.444	20.722	40.92	4.06	Low	NAT	P	13.78
LiNP	Chilopoda	Lithobiomorpha	Lithobiidae	*Lithobius*	*pilicornis*	37.092	23.947	55.693	10.44	Low	NAT	P	21.83
JuIS	Diplopoda	Julida	Julidae	*Cylindroiulus*	*propinquus*	25.459	20.639	34.504	10.68	Low	EXO	S	37.44
JuIH	Diplopoda	Julida	Julidae	*Ommatoiulus*	*moreletii*	40.839	27.478	64.985	9.44	Low	EXO	H	40.04
PoIS	Diplopoda	Polydesmida	Polydesmidae	*Polydesmus*	*coriaceus*	60.879	23.195	64.985	4.18	Low	EXO	S	25.38
CoEP	Insecta	Coleoptera	Carabidae	*Cedrorum*	*azoricus*	17.178	17.178	20.722	9.27	Low	END	P	10.71
CoEP	Insecta	Coleoptera	Carabidae	*Trechus*	*terrabravensis*	17.178	17.178	24.235	7.85	Low	END	P	2.58
CoEH	Insecta	Coleoptera	Curculionidae	*Drouetius*	*borgesi*	26.13	20.556	37.092	7.16	Low	END	H	8.12
CoEH	Insecta	Coleoptera	Elateridae	*Alestrus*	*dolosus*	23.615	17.178	24.982	6.08	Low	END	H	5.01
CoNP	Insecta	Coleoptera	Staphylinidae	*Ocypus*	*aethiops*	47.901	24.434	60.879	9.51	Low	NAT	P	9
HeIH	Insecta	Hemiptera	Aphididae	*Rhopalosiphonimus*	*latysiphon*	17.937	17.178	24.235	7.39	Low	EXO	H	1.18
HeEH	Insecta	Hemiptera	Cicadellidae	*Aphrodes*	*hamiltoni*	25.459	21.332	40.839	12.68	Low	END	H	2.31
HeEH	Insecta	Hemiptera	Cixiidae	*Cixius*	*azoterceirae*	25.459	21.319	37.092	11.29	Low	END	H	5
HeNH	Insecta	Hemiptera	Delphacidae	*Megamelodes*	*quadrimaculatus*	24.687	17.178	40.839	5.53	Low	NAT	H	3
HeNH	Insecta	Hemiptera	Flatidae	*Cyphopterum*	*adcendens*	23.352	17.937	24.982	8.49	Low	NAT	H	5
LeIH	Insecta	Lepidoptera	Tortricidae	*Gen*	*sp.*	25.459	21.684	37.092	12.78	Low	EXO	H	16.41
LeEH	Insecta	Lepidoptera	Yponomeutidae	*Argyresthia*	*atlanticella*	31.34	21.006	42.458	9.5	Low	END	H	3.44
MiES	Insecta	Microcoryphia	Machilidae	*Trigoniophthalmus*	*borgesi*	25.459	22.585	34.504	4.67	Low	END	S	14.11

ArIP	Arachnida	Araneae	Gnaphosidae	*Zelotes*	*aeneus*	63.922	57.274	71.143	14.87	High	EXO	P	6
ArIP	Arachnida	Araneae	Linyphiidae	*Erigone*	*autumnalis*	39.022	26.95	63.037	12.48	High	EXO	P	2.2
ArIP	Arachnida	Araneae	Linyphiidae	*Erigone*	*dentipalpis*	37.092	26.909	60.879	13.17	High	EXO	P	2.3
ArIP	Arachnida	Araneae	Linyphiidae	*Meioneta*	*fuscipalpa*	66.427	55.093	71.143	15.66	High	EXO	P	1.9
ArIP	Arachnida	Araneae	Linyphiidae	*Mermessus*	*bryantae*	39.022	24.982	55.093	6.29	High	EXO	P	2.2
ArIP	Arachnida	Araneae	Linyphiidae	*Mermessus*	*fradeorum*	44.86	26.13	63.341	7.85	High	EXO	P	2.2
ArIP	Arachnida	Araneae	Linyphiidae	*Oedothorax*	*fuscus*	39.022	25.459	63.037	11.74	High	EXO	P	2.75
ArIP	Arachnida	Araneae	Linyphiidae	*Ostearius*	*melanopygius*	55.693	37.092	66.011	13.07	High	EXO	P	2.3
ArIP	Arachnida	Araneae	Linyphiidae	*Prinerigone*	*vagans*	57.274	32.734	63.922	13.06	High	EXO	P	2.1
ArIP	Arachnida	Araneae	Tetragnathidae	*Pachygnatha*	*degeeri*	55.693	29.033	64.985	11.44	High	EXO	P	3.38
OpNP	Arachnida	Opiliones	Phalangiidae	*Homalenotus*	*coriaceus*	23.947	20.722	34.504	4.47	High	NAT	P	3.82
LiNP	Chilopoda	Lithobiomorpha	Lithobiidae	*Lithobius*	*sp.*	57.274	40.839	66.011	12.65	High	NAT	P	8.97
CoNS	Insecta	Coleoptera	Anthicidae	*Hirticollis*	*quadriguttatus*	64.985	52.443	70.804	14.38	High	NAT	S	2.61
CoIP	Insecta	Coleoptera	Carabidae	*Amara*	*aenea*	57.274	44.197	67.219	9.59	High	EXO	P	7
CoIP	Insecta	Coleoptera	Carabidae	*Anisodactylus*	*binotatus*	43.174	24.235	60.879	6.47	High	EXO	P	11
CoNP	Insecta	Coleoptera	Carabidae	*Calosoma*	*olivieri*	40.839	29.033	66.011	9.84	High	NAT	P	23
CoIU	Insecta	Coleoptera	Carabidae	*Pseudophonus*	*rufipes*	39.022	26.13	63.922	7.44	High	EXO	U	13
CoIP	Insecta	Coleoptera	Carabidae	*Pterostichus*	*vernalis*	29.033	24.444	44.197	6.2	High	EXO	P	7
CoIH	Insecta	Coleoptera	Chrysomelidae	*Chaetocnema*	*hortensis*	57.274	44.197	66.011	13.74	High	EXO	H	2.01
CoNH	Insecta	Coleoptera	Chrysomelidae	*Psylliodes*	*marcidus*	70.804	43.797	71.143	12.2	High	NAT	H	2.56
CoIP	Insecta	Coleoptera	Corylophidae	*Sericoderus*	*lateralis*	66.427	44.11	71.143	8.89	High	EXO	P	0.5
CoIS	Insecta	Coleoptera	Cryptophagidae	*Cryptophagus*	*sp.*	60.879	34.504	70.804	9.84	High	EXO	S	1.44
CoIS	Insecta	Coleoptera	Cryptophagidae	*Cryptophagus*	*sp.*	64.985	44.197	71.143	7.22	High	EXO	S	2.2
CoIH	Insecta	Coleoptera	Curculionidae	*Sitona*	*discoideus*	57.274	27.451	63.922	8.35	High	EXO	H	5.79
CoIH	Insecta	Coleoptera	Curculionidae	*Sitona*	*sp.*	44.197	32.734	63.037	10.76	High	EXO	H	4.27
CoIH	Insecta	Coleoptera	Dryophthoridae	*Sphenophorus*	*abbreviatus*	44.86	24.982	63.037	7.1	High	EXO	H	10.45
CoES	Insecta	Coleoptera	Elateridae	*Heteroderes*	*azoricus*	63.037	52.443	70.804	16.1	High	END	S	7.65
CoIS	Insecta	Coleoptera	Elateridae	*Heteroderes*	*vagus*	55.093	44.197	69.284	9.71	High	EXO	S	6.55
CoIF	Insecta	Coleoptera	Mycetophagidae	*Typhaea*	*stercorea*	63.037	44.86	69.284	15.6	High	EXO	F	2.27
CoIS	Insecta	Coleoptera	Nitidulidae	*Epuraea*	*biguttata*	29.033	23.352	66.011	4.33	High	EXO	S	2.98
CoIS	Insecta	Coleoptera	Phalacridae	*Gen.*	*sp.*	44.86	26.95	71.143	5.56	High	EXO	S	1.34
CoIP	Insecta	Coleoptera	Staphylinidae	*Aleochara*	*bipustulata*	57.274	27.478	70.804	8.1	High	EXO	P	3
CoIP	Insecta	Coleoptera	Staphylinidae	*Amischa*	*analis*	57.274	24.687	65.036	9.97	High	EXO	P	3
CoIP	Insecta	Coleoptera	Staphylinidae	*Cordalia*	*obscura*	40.839	29.033	64.985	13.03	High	EXO	P	2
CoNP	Insecta	Coleoptera	Staphylinidae	*Ocypus*	*olens*	34.504	26.13	69.36	4.81	High	NAT	P	17

CoIP	Insecta	Coleoptera	Staphylinidae	*Oligota*	*parva*	67.219	43.138	71.143	10.29	High	EXO	P	1
CoNP	Insecta	Coleoptera	Staphylinidae	*Rugilus*	*orbiculatus*	40.839	25.459	63.341	9.8	High	NAT	P	4
CoIP	Insecta	Coleoptera	Staphylinidae	*Xantholinus*	*longiventris*	29.033	26.95	63.341	7.17	High	EXO	P	7
DeIP	Insecta	Dermaptera	Forficulidae	*Forficula*	*auricularia*	66.427	42.377	69.284	13.64	High	EXO	P	16
HeNH	Insecta	Hemiptera	Cicadellidae	*Euscelidius*	*variegatus*	60.879	39.022	67.219	13.43	High	NAT	H	3.56
HeNP	Insecta	Hemiptera	Nabidae	*Nabis*	*pseudoferus*	55.693	34.504	66.427	10.07	High	NAT	P	9
LeNH	Insecta	Lepidoptera	Noctuidae	*Mythimna*	*unipuncta*	37.092	24.967	60.879	11.12	High	NAT	H	21.01
LeEH	Insecta	Lepidoptera	Noctuidae	*Phlogophora*	*interrupta*	29.033	25.459	71.143	4.58	High	END	H	40
OrIS	Insecta	Orthoptera	Gryllidae	*Gryllus*	*bimaculatus*	66.427	57.274	71.143	12.69	High	EXO	S	32.69
